# The WHO Global Code: increasing relevance and effectiveness

**DOI:** 10.1186/s12960-016-0131-x

**Published:** 2016-06-30

**Authors:** James Campbell, Ibadat S. Dhillon, Amani Siyam

**Affiliations:** Health Workforce Department, World Health Organization, Geneva, Switzerland; Global Health Workforce Alliance (GHWA), Geneva, Switzerland; Information, Evidence and Research Department, World Health Organization, Geneva, Switzerland

The international migration of health personnel is on the rise. Recent data demonstrate increasing reliance on foreign-born and foreign-trained health professionals in OECD countries with a 60 % increase in the total number of migrant nurses and physicians over the last decade and a higher proportionate increase from countries experiencing ‘critical’ health workforce shortages [1]. These trends are projected to worsen [2]. OECD countries are not the only destination. Emerging economies in Asia, the Middle East, and sub-Saharan Africa are increasingly the destinations for migrating health professionals [3].

The WHO Global Code of Practice on the International Recruitment of Health Personnel (hereafter ‘the Code’) is an instrument of important normative value [4]. Adopted by all Member States of the WHO, the Code establishes and promotes voluntary principles and practices to better manage the international migration of health personnel, with a focus on strengthening health systems. In 2015, the WHO Executive Board requested the DG to conduct the first review of the relevance and effectiveness of the Code [5]. An independent Expert Advisory Group (EAG) was established and following deliberations concluded that the Code remains ‘highly’ relevant, particularly in the context of increasing intra and inter-regional labour mobility. The EAG also identified implementation-related weakness and provided specific recommendations, namely:

- a call for countries to advance awareness and implementation of the Code;
- a call on the WHO secretariat to expand its capacity to provide global, regional, and country level support to advance effective implementation of the Code; and
- a request for WHO to reassess the Code’s relevance and effectiveness in line with the third round of national reporting in 2019.

WHO Member States completed the second round of national reporting with significant improvement in both quantity and quality [3]. There has been a 37 % increase in countries identifying national designated authorities and the number of countries submitting national reports increased by 32 %. In collaboration with OECD and Eurostat, a module was developed and provided in the second round to assist countries reporting on health workforce migration data allowing good insight into mobility patterns. Across the various national reports, there has been a systematic call for technical assistance towards the development of national regulation; support for better linking international agreements and national regulations; and for funding to standardize, collect and exchange health labour mobility data.

The fourteen papers included in this special supplement add to the evidence on the relevance and effectiveness of the Code, and propose actions in support of the EAG recommendations. A central point captured across the papers is the striking lack of systematically collected data on health personnel migration in neither countries of origin nor destination. Undoubtedly a major obstacle to the action and coordination that is required. Paina et al. [6], in particular, identify data collection and information as the two cornerstones of the Code. They also speak to the urgent need to make international bilateral agreements transparent and accessible, a concern raised previously by others [7].

Two of the papers, Abugala and Badr [8] and Abadalla et al. [9], speak to the ‘unprecedented’ exodus of skilled health professionals from Sudan. They identify a ten-fold increase in the request of certificates for good standing, a proxy for the intention to emigrate, between 2000 and 2013. This highlights the importance of capturing ‘exit’ data, with the magnitude of emigration from low income countries likely to be significantly higher than currently estimated. Abugala and Badr [8] are particularly concerned by Sudan’s loss of academic staff and its consequent impact on the reduced training capacity for nursing, midwifery and allied health workers. They speak favourably to the role of the Code in galvanizing action at the national level, including the signing of bilateral agreements with destination countries. Yet they also critique the voluntary nature of the Code, particularly alongside limited civil society activism in destination countries.

A number of papers stress the importance of low salaries, poor working conditions, and poor career progression as push factors for emigration [9–12]. These same factors are also explicitly identified, alongside concerns of stability and security, as inhibiting return migration. Insofar as circular migration is a potential policy response, Abdalla et al. [9], Poppe et al. [10], and Tomblin-Murphy et al. [11], identify significant reluctance amongst the diaspora to return for medium to longer periods. The findings raise an important question in terms of the value of short-term return migration, as contrasted with the administrative and financial costs to the country of origin.

Tyrell et al. [13] and Brugha et al. [14] add important insight on the value of career progression for retaining health workers. Contrary to the Code’s recommendation on equality, Tyrell et al. found that physicians trained outside of Ireland were significantly less likely to experience career progression than those trained in Ireland [12]. Brugha et al. [14] found that approximately half of foreign doctors intended to migrate onwards. Most of them pointed to a lack of career progression, with two-thirds planning to return home having failed to gain entry in a post-graduate training scheme [14]. Both Tyrell et al. and Brugha et al. point to the need for close scrutiny on medical workforce policies [13, 14]. Hitherto, Brugha et al. note that the adoption of the Code did instigate such examination at the national level [14]. On another note, McAleese et al. [15] warn that the longer health professionals remain abroad, the less likely are they to return to their home countries.

In contrast to other papers, Laytin and Derbew [16], point to a success story of retention. Over three-quarters of all Ethiopian surgeons trained nationally (and in Cuba) were still working in Ethiopia, with a majority in the public sector. Potential explanations for this are linked to the ability to engage in dual practice and the availability of sub-specialty training. Closer examination of Ethiopia’s particular context is necessary to build upon Ethiopia’s success.

Squires et al. [17], present exam test results for registered nurses to practice in the US over the last decade, linking accreditation and licensing processes, international migration, and health workforce education in destination countries. They identify that the pass rates on the US Nurse Credentialing and Licensure Examination have remained the same (approximately 87 % in the period 2003 to 2014), despite significant changes to the test in 2005, 2008, 2011, and 2014. In the same period, however, the pass rates of foreign-trained nurses (primarily from India and the Philippines) has fallen consistently from 58.1 % in 2005 to below 30 % in 2014. The study opens the question of the ethics around modification of nursing curricula in countries seeking to export nurses, as well as that questions of quality of training in countries without stringent accreditation and licensure processes.

Bourgeault et al. and Van de Pas et al. examined the overall relevance and effectiveness of the Code [18, 19]. Bourgeault et al. in particular identify a significant gap in knowledge of the Code and also point to the lack of enforceability of the Code as a central weakness [18]. Van de Pas et al., on the other hand, make an important distinction between the Code’s effectiveness in Europe and that in East and Southern Africa [19]. While they find evidence within the European context of the Code impacting practice at the national and local level, they were not able to in East and Southern Africa. The main challenges were identified as lack of coordinated and comprehensive data, lack of shared understanding between stakeholders, lack of information exchange, and a lack of civil society voice. They went further to suggest that definitions within the Code be further clarified and that a new governance structure be instituted. In particular, Van de Pas et al. [19] recommended that a Global Health Resource Fund, loosely based on UNITAID [20], be established. They call for a dynamic fee structure and obligatory payments from high income countries and private sector actors to contribute to health system strengthening and health employment funding.

Finally, Schaffer et al. [21] provide an important illustration of how the principles of the Code can be advanced through the development of governance-related processes at national and regional levels. The CGFNS Alliance Code, developed prior to but linking to the Code recommendations, brought together recruiters, employers, nursing organizations, unions, and researchers. This bottom up approach enabled a strong, detailed, and contextually relevant framework to better manage health professional migration within the US context. However, Schaffer et al. do caution that a strong governance structure with appropriate financing is needed to ensure success of such national and local models [21].

At the global level, the Global Strategy on Human Resources for Health: Workforce 2030 signals an increasing mismatch between the global supply, economic demand, and need for health workers [2]. This mismatch is likely to exacerbate the negative consequences of international mobility, despite the Code being an integral and indivisible part of the Global Strategy. Health labour markets are interconnected and inter-dependent. Intersectoral and political engagement at the highest level are imperative if countries are to deliver on the SDG agenda. In March 2016, the UN Secretary-General announced the High-Level Commission on Health Employment and Economic Growth. In its first communique the Commission included a particular focus on addressing the negative effects associated with the international mobility of health personnel. The collective evidence of this supplement will therefore serve to inform the Commission’s deliberations and recommendations, reaffirming that the Code remains a relevant and effective framework.
